# Intra-abdominal Adhesions among Patients Undergoing Repeat Caesarean Section in Department of Obstetrics and Gynaecology of a Tertiary Care Centre: A Descriptive Cross-sectional Study

**DOI:** 10.31729/jnma.7547

**Published:** 2022-06-30

**Authors:** Manoj Pokhrel, Lakpa Dolma Sherpa, Meena Thapa, Jyotshna Sharma

**Affiliations:** 1 Department of Obstetrics and Gynaecology, Kathmandu Medical College and Teaching Hospital, Sinamagal, Kathmandu, Nepal

**Keywords:** *postoperative complications*, *repeat caesarean section*, *surgical adhesions*

## Abstract

**Introduction:**

Adhesions are one of the common complications encountered after caesarean section whose risk increases with the number of caesarean deliveries. This study aimed to find out the prevalence of intra-abdominal adhesions among patients undergoing repeat caesarean section in a tertiary care centre.

**Methods:**

A descriptive cross-sectional study was conducted on 74 pregnant women undergoing repeat caesarean section in the Department of Obstetrics and Gynaecology of a tertiary care centre from July, 2021 to December, 2021 after receiving the ethical approval from the Institutional Review Committee (Reference number: 2107202103). Pregnant women who met the eligibility criteria were included in the study. Convenience sampling was done. The severity of the adhesions was classified using the Tulandi and Lyell classification. Data were analysed using the Statistical Package for the Social Sciences version 26.0 software. Point estimate at 90% Confidence Interval was calculated along with frequency and percentage for binary data along with mean and standard deviation for continuous data.

**Results:**

Out of 74 women undergoing repeat caesarean section, 55 (74.32%) (65.99-82.65 at 90% Confidence Interval) had developed intra-abdominal adhesions.

**Conclusions:**

Our study showed that the prevalence of intra-abdominal adhesions among patients undergoing repeat caesarean section was higher when compared to similar studies conducted in similar settings.

## INTRODUCTION

Caesarean Section (CS) is a common abdominal surgical procedure that has been increasing worldwide.^1,2^ Adhesions are one of the complications seen after CS that can result in subfertility, chronic pain, intestinal obstruction, and other complications such as bladder and bowel injury, blood loss, and prolonged operating time in subsequent surgeries.^3-7^

Studies have shown that, in women with a history of one previous CS, adhesions could be seen in 24% to 65%, which increases with the number of caesarean deliveries.^7-10^

This study aimed to find out the prevalence of intraabdominal adhesions among patients undergoing repeat caesarean section in a tertiary care centre.

## METHODS

A descriptive cross-sectional study was conducted on 74 pregnant women undergoing repeat caesarean section in the Department of Obstetrics and Gynaecology of Kathmandu Medical College and Teaching Hospital from July, 2021 to December, 2021 after receiving the ethical approval from the Institutional Review Committee (Reference number: 2107202103). Pregnant women undergoing elective or emergency section with history of previous caesarean section irrespective of number and type of CS and history of any other abdominal surgeries were excluded. Women undergoing caesarean section for the first time were excluded. Convenience sampling was done and the sample size was calculated using the formula,


$$\begin{array}{ll} \text{n} & ={\text{Z}}^{2}\times \frac{\text{p}\times \text{q}}{{\text{e}}^{\text{2}}}\\ & =1.64^{2}\times \frac{0.38\times 0.62}{0.1^{2}}\\ & =64 \end{array}$$

Where,

n = minimum required sample size
Z = 1.64 at 90% Confidence Interval (CI)
p = prevalence of intra-abdominal adhesions among patients undergoing repeat caesarean section, 38%^11^
e = margin of error, 10%

Therefore, the minimum required sample size was 64. However, a sample size of 74 was taken. The severity of the adhesions was classified using the Tulandi and Lyell classification.^12^ Data regarding the presence of adhesions, severity of adhesions, age of the patients, gravida of the patients, history of prior intra abdominal surgeries and the location of the adhesions were collected. Data were entered and analysed using the Statistical Package for the Social Sciences version 26.0 software. Point estimate at 90% Confidence Interval was calculated along with frequency and percentage for binary data along with mean and standard deviation for continuous data.

## RESULTS

Out of 74 women undergoing repeat caesarean section, 55 (74.32%) (65.99-82.65 at 90% Confidence Interval) had developed intra-abdominal adhesion. The adhesion was mild in 51 (92.72%) and severe in 4 (7.27%) cases according to Tulandi and Lyell Classification.

The mean incision to delivery time was 9±6.23 minutes during caesarean section with adhesions which was 15.50±5.28 minutes in severe adhesions (Table 1).

**Table 1:** Incision to delivery time according to the grading of adhesion (n = 55).

Grade of adhesion	Mean±SD (minutes)
Mild adhesion	8.47±5.08
Severe adhesion	15.50±7.14

The operating time was 51.65±19.61 minutes in mild adhesions, and 75±20.44 minutes in severe adhesion (Table 2).

**Table 2:** Total operating time according to the grading of adhesion (n = 55)

Grade of adhesion	Mean±SD (minutes)
Mild adhesion	51.65±19.61
Severe adhesion	75.00±33.42

Regarding the location, the intra-abdominal adhesions was most commonly seen between the uterus and bladder in 23 (41.82%) (Table 3).

**Table 3:** Location of adhesions among patients undergoing repeat caesarean section (n = 55).

Location of adhesions	n (%)
Uterus and bladder	23 (41.82)
Uterus and bladder, uterus and fascia	12 (21.82)
Uterus and bladder, omentum and fascia	7 (12.73)
Uterus and bladder, uterus and fascia, uterus and omentum	4 (7.27)
Uterus and bladder, uterus and fascia, uterus and omentum, omentum and fascia	2 (3.63)
Uterus and fascia	2 (3.63)
Uterus and omentum	2 (3.63)
Uterus and bladder, uterus and omentum	1 (1.82)
Uterus and bladder, uterus and omentum, omentum and fascia	1 (1.82)
Omentum and fascia	1 (1.82)

Fifty-one (92.72%) women with adhesions had history of one previous CS, and 4 (7.27%) women had two or more prior CS and/or previous pelvic or abdominal surgery. Intraoperative complications noted during CS were scar dehiscence in 6 (8.10%).

## DISCUSSION

This study found out that the majority of women with a history of CS had intra abdominal adhesions which could have affected the incision to the delivery time and the operative time. The prevalence and severity of the intraabdominal adhesions increased with the number of prior intra abdominal surgeries.

These findings are comparable with other studies which have shown that adhesions are a frequent complication encountered after CS.^7,8^ The prevalence of intra-abdominal adhesion in our study was higher than a similar study.^11^ This study also reported that the prevalence of adhesions increased with a history of CS and resulted in prolonged operative time which was consistent with the findings of our study.

Significant association of CS with adhesions was shown by a study which included patients who underwent various prior abdominopelvic surgery and subsequently underwent gynaecological laparoscopic surgery for various indications. The intra abdominal adhesions were present in 36.1% of the patients which was lower when compared to our study.^13^ A study reported that 45.1% of patients with a history of CS showed evidence of pelvic adhesions during the ultrasonographic examination.^14^ Our study had shown that the mean operating time was higher in severe adhesions than mild adhesions which was comparable to another similar study.^11^ In this study the commonest adhesion was present between the uterus and urinary bladder (41.82%) which is consistent with another similar study.^15^

The key limitations of this study include the small sample size and single-centre nature which can reduce the generalizability of the findings. The cross-sectional design of the study did not include follow-up of the patients. Higher studies could be done to study the causality and factors associated with adhesions including the technique of surgery.

## CONCLUSIONS

Our study showed that the prevalence of intra-abdominal adhesions among patients undergoing repeat caesarean section was higher when compared to similar studies conducted in similar settings. The morbidity could increase with the number of repeat caesarean sections. It is paramount that the rate of primary caesarean sections should be reduced in order to prevent maternal and foetal morbidities. With the increasing global trend of caesarean section in general, pregnant women need proper counselling with regards to their options and associated short- and long-term consequences.
